# Human MAIT cells show metabolic quiescence with rapid glucose‐dependent upregulation of granzyme B upon stimulation

**DOI:** 10.1111/imcb.12020

**Published:** 2018-03-09

**Authors:** Madeleine E Zinser, Andrew J Highton, Ayako Kurioka, Barbara Kronsteiner, Joachim Hagel, Tianqi Leng, Emanuele Marchi, Chansavath Phetsouphanh, Chris B Willberg, Susanna J Dunachie, Paul Klenerman

**Affiliations:** ^1^ Peter Medawar Building for Pathogen Research Nuffield Department of Medicine University of Oxford Oxford United Kingdom; ^2^ Centre for Tropical Medicine and Global Health Nuffield Department of Medicine University of Oxford Oxford United Kingdom; ^3^ National Institute for Health Research Oxford Biomedical Research Centre University of Oxford Oxford United Kingdom

**Keywords:** MAIT cells, metabolism, mucosal immunology, T cells

## Abstract

Mucosal‐associated invariant T (MAIT) cells are a well‐characterized innate‐like T cell population abundant in the human liver, peripheral tissues and blood. MAIT cells serve in the first line of defense against infections, through engagement of their T cell receptor, which recognizes microbial metabolites presented on MR1, and through cytokine‐mediated triggering. Typically, they show a quiescent memory phenotype but can undergo rapid upregulation of effector functions including cytolysis upon stimulation. T cells profoundly change their cellular metabolism during their maturation and activation. We sought to determine how MAIT cell metabolism may facilitate both the long‐term memory phase in tissue and the transition to rapid effector function. Here, we show, by flow cytometric metabolism assays and extracellular flux analysis that, despite an effector‐memory profile, human MAIT cells are metabolically quiescent in a resting state comparable to naïve and central memory T cells. Upon stimulation, they rapidly increase uptake of glucose and show a concomitant upregulation of the effector molecules notably granzyme B, which is impaired by inhibition of glycolysis with 2‐deoxyglucose. These findings suggest that MAIT cells share some metabolic characteristics of both resting and effector T cell subsets, with a rapid transition upon triggering. Metabolic programming of this cell type may be of interest in understanding and modulating their function in infectious diseases and cancer.

## Introduction

Mucosal‐associated invariant T (MAIT) cells constitute a population of innate‐like T cells abundant in humans. They make up to 1–10% of peripheral T cells and up to 50% of hepatic T cells.^1^ They are also present at mucosal surfaces^2^ and have been shown to play important roles in settings of bacterial^3^ and viral infection^4^ as well as cancer.^5^ They express a semi‐invariant T cell receptor (Vα7.2) on their cellular surface,^6^ through which they recognize ligands presented by the nonclassical MHC class Ib molecule MR‐1 (MHC‐class I related protein 1).^2^ MR‐1 binds metabolites of the riboflavin metabolism pathway, found in certain bacteria such as *Escherichia coli*. Combined staining for both T cell receptor (TCR) Vα7.2 (or MR1‐ligand tetramer) and CD161 allows MAIT cells to be distinguished from other T cell populations. They possess an effector‐memory phenotype,^1^ do not express CCR7 and are either CD45RA positive or negative. Upon stimulation through TCR ligation or by cytokine‐driven activation (IL‐12 and IL‐18),^7^ they rapidly carry out their effector functions by secreting IFN‐γ, IL‐17, granzyme B, perforin and TNF‐α.^1^, ^7^, ^8^, ^9^ Due to their location in tissues frequently exposed to pathogens (blood, intestine and liver), they serve as a rapid bridging response. Immune cells differ in their cellular metabolism according to their activation state. Recognition of cognate antigen during an immune response by naïve T cells switches them from a metabolically quiescent state, preferentially using oxidative phosphorylation for ATP production, to a more active state, predominantly reliant upon the use of aerobic glycolysis.^10^ Once the antigen is cleared, “metabolically primed” memory T cells revert to oxidative phosphorylation and fatty acid oxidation.^11^ Similarly, innate responses show preferential use of glycolysis and the utilization of the pentose phosphate pathway in acute responses.^12^

Due to their specific localization and their dual mode of action—long term surveillance *versus* rapid activation—MAIT cells need to adapt their metabolism accordingly. In this study, we provide the first evidence of metabolic properties of MAIT cells by integrating gene expression and functional data. Our data show that MAIT cells, similar to naïve T cells or central memory cells are metabolically quiescent in the resting state. Upon stimulation, MAIT cells preferentially upregulate their glycolytic activity and this upregulation is accompanied by enhanced expression of the effector molecule granzyme B.

## Results

### Transcriptional analysis reveals a distinct pattern of metabolic gene transcript sets in CD161^++^ CD8^+^ T cells

For gene expression analysis, we used a microarray dataset on sorted CD161^++^, CD161^+^ and CD161^−^ CD8^+^ T cells from four different healthy blood donors that was previously published by our group.^8^ Of note, the human peripheral CD161^++^ CD8α^+^ T cell pool largely consists of MAIT cells, making up to 90% of this population,^9^ with the rest showing a very similar transcriptional and functional profile. We performed Gene Set Enrichment Analysis^13^ on predefined metabolic gene sets from the KEGG (Kyoto Encyclopedia of Genes and Genomes) database for multiple metabolic pathways including glycolysis and oxidative phosphorylation. This analysis revealed that most metabolic gene sets, including glycolysis and oxidative phosphorylation, are enriched in the control CD161^−^ CD8^+^ population (i.e. downregulated in the CD161^++^ cells) and only gene transcripts relevant for galactose metabolism were enriched in the CD161^++^ CD8^+^ population (Supplementary figure 1a). The normalized enrichment scores for transcripts relevant for oxidative phosphorylation and the glycolytic pathway were −1.20 and −1.09, respectively (Supplementary figure 1b, c). Leading edge transcripts of Gene Set Enrichment Analysis of oxidative phosphorylation are represented in Supplementary figure 1d. Individual comparisons of gene set enrichment between CD161^hi^ and CD161^lo^ *T*_EM_ and *T*_CM_ 14 memory subpopulations and between CD161^hi^ *T*_EM_ and naïve CD8^+^ T cells are represented in Supplementary figure 1e. Taken together, these data point toward a differential expression of important key metabolic transcripts in resting CD161^++^ CD8^+^ T cells compared to the more heterogeneous population of CD161^−^ CD8^+^ T cells consisting of CD161^−^ naïve (*T*_N_: 16.85%; IQR 9.6–25.47%), central memory (*T*_CM_: 6.25%; IQR 3.83–8.08%), effector memory (*T*_EM_: 24.67%; IQR 16.53–32.98%) and terminally differentiated memory cells (*T*_EMRA_: 51.98%; IQR 41.73–58.1%). This suggests that peripheral CD161^++^ CD8^+^ T cells exist in a more quiescent state compared to CD161^−^ CD8^+^ T cells.

### Low mitochondrial mass and mitochondrial depolarization in CD161^++^/MAIT cells is distinct from CD8^+^ *T*_EM_ subsets

**Figure 1:**
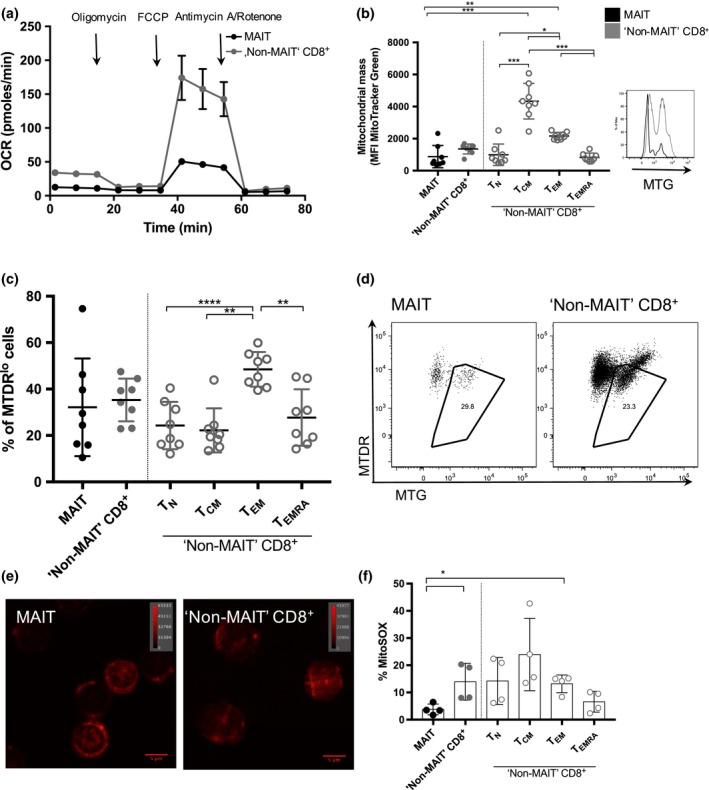
Decreased “mitochondrial activity” in MAIT cells. **(a)** Oxygen consumption rate (OCR) obtained during mitochondrial stress test, performed by injection of oligomycin, FCCP, antimycin A and rotenone showing decreased spare respiratory capacity (SRC) in MAIT cells *versus* “Non‐MAIT” CD8^+^ T cells. Sorted MAIT and “Non‐MAIT” CD8^+^ T cells were used from one healthy blood donor with a peripheral MAIT cell percentage of 32.8% of CD8^+^ T cells. **(b)** MitoTracker Green MFI of MAIT cells *versus* “Non‐MAIT” CD8^+^ T cells. “Non‐MAIT” T cells are further divided into the subsets *T*_N_,*T*_CM_,*T*_EM_ and *T*_EMRA_ (left). Significance was determined using a paired, parametric two‐tailed *t*‐test. Significance levels: * indicates *P* < 0.05, **P < 0.01, ***P < 0.001. Error bars show mean ± s.d. Data for *n* = 8 healthy donors are shown (representative of three independent experiments). Representative histogram for one donor showing staining for MitoTracker Green (MTG) staining (right). **(c)** MAIT cells *versus* “Non‐MAIT” CD8^+^ T cells (constituted of *T*_N_,*T*_CM_,*T*_EM_ and *T*_EMRA_) with depolarized mitochondria identified as positive for MTG and expressing low levels of MitoTracker DeepRed (MTDR ^lo^). Data for *n* = 8 healthy donors are shown (representative of three independent experiments). **(d)** Representative gating strategy for one donor for cells containing depolarized mitochondria showing both MAIT cells and “Non‐MAIT” CD8^+^ T cells. **(e)** Confocal image showing sorted MAIT cells (left) and “Non‐MAIT” CD8^+^ T cells (right) stained for MitoTracker DeepRed. Data from one healthy donor are shown. The magnification applied is 63× manually and further 3.6× using ZEN black software (Zeiss). The indicated lookup table is linear and covers the full range of the data. **(f)** Mitochondrial production of reactive oxygen species (ROS) measured by frequency of MitoSOX positive cells comparing MAIT cells and “Non‐MAIT” CD8^+^ T cells. “Non‐MAIT” CD8^+^ T cells were further subdivided into *T*_N_,*T*_CM_,*T*_EM_ and *T*_EMRA_. Significance was determined using a paired, parametric two‐tailed *t*‐test. Significance levels: * indicates *P* < 0.05, **P < 0.01, ***P < 0.001. Error bars show mean ± s.d. Data are shown from *n* = 4 healthy donors (representative of two independent experiments).

Mitochondria are the site of oxidative phosphorylation in a cell.^15^ Firstly, we assessed the dynamics of mitochondrial respiration using sorted MAIT (CD161^++^Vα7.2^+^ CD8^+^ T cells) and control (CD161^−^Vα7.2^−^ CD8^+^ T cells) cells by extracellular flux analysis using a Seahorse Mitochondrial Stress Test.^16^ Changes in oxygen consumption rate upon the addition of carbonyl cyanide‐p‐trifluoromethoxyphenylhydrazone, an agent that disrupts ATP synthesis by blocking the proton gradient across the mitochondrial membrane indicate that MAIT cells have a low maximal respiratory capacity (Maximal oxygen consumption rate; Figure 1a). This results in much lower spare respiratory capacity, which is expressed by the difference in maximal respiration after addition of carbonyl cyanide‐p‐trifluoromethoxyphenylhydrazone and respiration after addition of oligomycin (Supplementary figure 2a).

One possible reason for a lowered SRC can be a reduced number of mitochondria.^11^ Therefore, we determined the mitochondrial mass of MAIT cells compared to other T cell subsets, as well as their polarization status and functionality. Staining with MitoTracker Green revealed that MAIT cells have significantly lower mitochondrial content compared to *T*_CM_ and *T*_EM_ (Figure 1b). This finding matches the tendency towards higher mitochondrial content seen in memory cell subsets^17^ and was further supported by significant differences in mitochondrial mass between the different memory subsets in conventional “Non‐MAIT” CD8^+^ T cells with *T*_CM_ containing most mitochondria (Figure 1b). MAIT cells show a tendency towards a lower mitochondrial content than their *T*_EM_ CD8^+^ counterparts and their mitochondrial mass was more comparable to the mitochondrial mass of naïve (*T*_N_) and terminally differentiated effector memory cells (*T*_EMRA_) (Figure 1b).

Depolarization of mitochondria was assessed by staining with MitoTracker DeepRed, which preferentially accumulates within healthy polarized mitochondria.^18^, ^19^ MAIT cells showed similar mitochondrial depolarization compared to most CD8^+^ T cells (Figure 1c, d) whereas *T*_EM_ showed a significantly higher frequency of cells containing depolarized mitochondria compared to other CD8^+^ memory T cell subsets (Figure 1c). These findings match the previously observed negative enrichment of metabolic transcripts relevant for oxidative phosphorylation (Supplementary figure 1b) in CD161^++^ CD8^+^ T cells.

Depolarization findings were confirmed in *ex vivo* PBMCs using an alternative dye, JC‐1, that specifically stains for depolarized mitochondria^20^ (Supplementary figure 2b). An increased abundance of healthy mitochondria within sorted MAIT cells compared to control cells was observed by confocal fluorescence microscopy and MitoTracker DeepRed staining (Figure 1e).

### Low production of reactive oxygen species and apoptosis and preserved autophagy in MAIT cells

Mitochondria are the most predominant cellular source of reactive oxygen species (ROS) and are involved in a major regulatory pathway of apoptosis induction.^21^ Thus, we were interested in whether MAIT cells differ in the amount of ROS they produce and if they were more apoptotic compared to the other cell types. Using a dye that specifically stains for ROS produced in mitochondria, we observed that the ROS production in the MAIT cell population was overall lower compared to “Non‐MAIT” CD8^+^ T cells (Figure 1f). Looking at mitochondrial ROS production within “Non‐MAIT” CD8^+^ T cells, *T*_EMRA_ show the lowest amounts of mitochondrial reactive oxygen species. The frequency of apoptotic cells was by trend lowest in MAIT cells. Within “Non‐MAIT” cells, *T*_EM_ and *T*_EMRA_ CD8^+^ T cells displayed a tendency toward higher apoptotic rates when stimulated with anti‐CD3/CD28 (Supplementary figure 2c).

Autophagy has been shown to be essential for the formation of memory CD8^+^ T cells^22^ and constitutes an important cellular degradation pathway that recycles cellular content and can liberate metabolites for survival.^23^ We observed that all CD8^+^ T cell subsets examined had increased frequencies of cells containing autophagic vacuoles upon stimulation. This was most pronounced in MAIT cells, *T*_N_ and *T*_CM_ (Supplementary figure 2d).

### Analysis of glycolytic capacity in *ex vivo* MAIT cells

**Figure 2:**
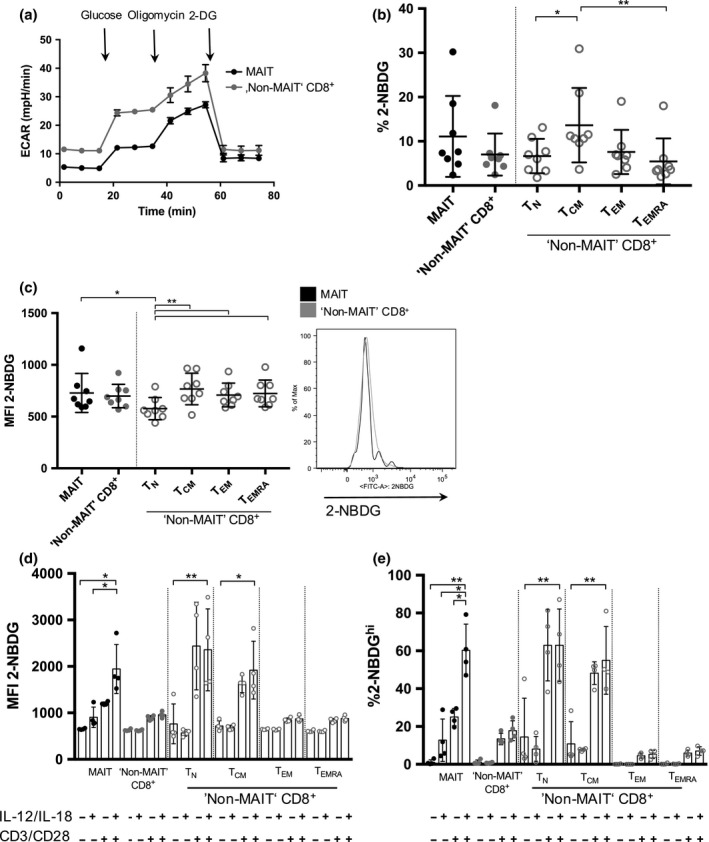
Glycolytic activity in MAIT cells and “Non‐MAIT” CD8^+^ T cells. **(a)** Glycolysis Stress Test measuring ECAR by injecting glucose to glucose‐starved cells, followed by oligomycin and 2‐deoxyglucose showing lower glycolytic capacity in MAIT cells compared to “Non‐MAIT” T cells. Sorted MAIT and “Non‐MAIT” CD8^+^ T cells were used from one healthy blood donor with a peripheral MAIT cell percentage of 21.2% of CD8^+^ T cells. **(b)** Uptake of 2‐NBDG measured in frequency of 2‐NBDG positive cells. CD161^−^ cells are further divided into *T*_N_,*T*_CM_,*T*_EM_ and *T*_EMRA__._ Cumulative data are shown (Tukey). Significance levels: * indicates *P* < 0.05, **P < 0.01, ***P < 0.001. Error bars show mean ± s.d. Cumulative data are shown for *n* = 8 healthy donors (representative of three independent experiments). **(c)** Uptake of 2‐NBDG measured in median fluorescent intensity of indicated cell subsets comparing MAIT cells and CD161^−^ CD8^+^ T cells (left) and representative histogram comparing MAIT cells and “Non‐MAIT” CD8^+^ T cells (right). CD161^−^ cells are further divided into *T*_N_,*T*_CM_,*T*_EM_ and *T*_EMRA__._ Cumulative data are shown for *n* = 8 healthy donors (representative of three independent experiments). **(d)** Uptake of 2‐NBDG measured in median fluorescent intensity of indicated cell subsets upon stimulation with the combination of cytokines IL‐12 and IL‐18 (50 ng mL^−1^) ± anti‐CD3/CD28. CD161^−^ cells were subdivided into *T*_N_,*T*_CM_,*T*_EM_ and *T*_EMRA_. Significance was determined using paired, parametric two‐tailed *t*‐test. Significance levels: * indicates *P* < 0.05, **P < 0.01, ***P < 0.001. Error bars show mean ± s.d. Cumulative data are shown for *n* = 4 healthy donors (representative of two independent experiments). **(e)** Uptake of 2‐NBDG measured in frequency of 2‐NBDG ^hi^ cells comparing MAIT cells and “Non‐MAIT” CD8^+^ T cells upon stimulation with the combination of cytokines IL‐12 and IL‐18 (50 ng mL ^−1^) ± anti‐CD3/CD28. CD161^−^ cells were subdivided into *T*_N_,*T*_CM_,*T*_EM_ and *T*_EMRA_. Significance was determined using a paired, parametric, two‐tailed *t*‐test. Significance levels: * indicates *P* < 0.05, **P < 0.01, ***P < 0.001. Error bars show mean ± s.d. Cumulative data are shown for *n* = 4 healthy donors (representative of two experiments).

As glycolysis was also transcriptionally downregulated in resting CD161^++^ CD8^+^ T cells (Supplementary figure 1c), we carried out a functional analysis examining glycolytic capacity in MAIT and “Non‐MAIT” CD8^+^ T cells by Seahorse Glycolysis Stress Test.^16^ Interestingly, the basal and maximal extracellular acidification rate was different between these two subsets (Figure 2a and Supplementary figure 2a). However, this difference was less pronounced compared to the difference in mitochondrial capacity of the two subsets. These findings are in line with a lower enrichment score in the glycolysis transcript set in CD161^−^ CD8^+^ T cells (Supplementary figure1c).

We next investigated the cellular uptake of the fluorescent glucose analogue 2‐NBDG (2‐(N‐(7‐Nitrobenz‐2‐oxa‐1,3‐diazol‐4‐yl) Amino)‐2‐Deoxyglucose). Its uptake was comparable in all examined cell types, both in terms of frequency of 2‐NBDG positive cells (Figure 2b) and the median fluorescence intensity (Figure 2c). There was no significant difference between MAIT cells and conventional T cells. Of these, *T*_N_ showed the least capacity for glucose uptake compared to all other examined subsets (Figure 2b,c), consistent with previous reports.^17^

### Upregulation of granzyme B in MAIT cells upon stimulation is dependent on glycolysis

Following optimal stimulation (TCR + cytokines), MAIT cells showed an increased uptake of the fluorescent glucose analogue 2‐NBDG (Figure 2d). This was most pronounced in MAIT cells as well as in only TCR‐stimulated *T*_N_ and *T*_CM_ CD8^+^ T cells. In contrast, in *T*_EM_ and *T*_EMRA_ no significant increase in glucose uptake was observed upon stimulation. The increased uptake of 2‐NBDG in MAIT cells occurred in the presence of IL‐12 and IL‐18 alone, CD3/CD28‐beads alone and synergistically in combination. After overnight stimulation and subsequent short‐term incubation with 2‐NBDG, most of the cells take up the fluorescent analogue independent of the cell type examined (data not shown). Thus, we decided to look at cells that take up high amounts of 2‐NBDG (2‐NBDG^hi^ cells) within each subset. By doing so, we could again observe an increased uptake of glucose. In *T*_N_ and *T*_CM_ cells, uptake was mostly dependent on TCR stimulation and not affected by cytokine stimulation, whereas in MAIT cells we could observe an upregulation of glucose uptake upon cytokine stimulation alone (Figure 2e).

**Figure 3:**
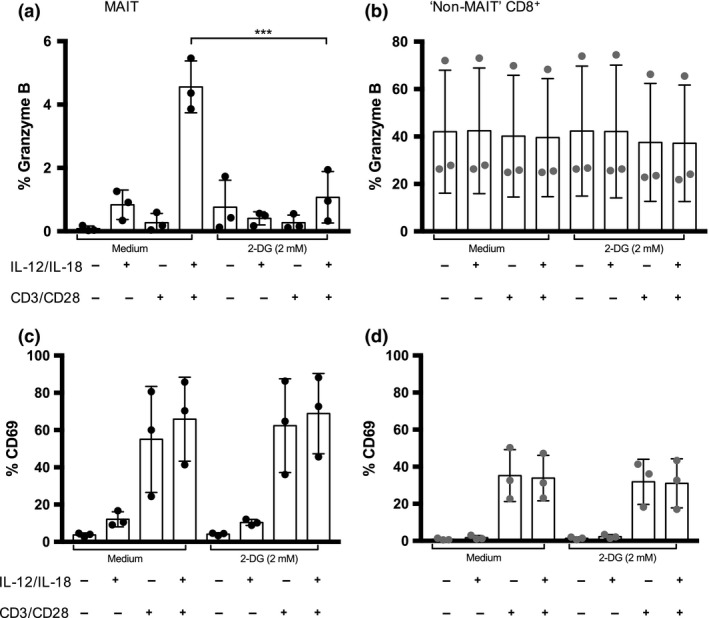
Upregulation of granzyme B in MAIT cells and “Non‐MAIT” CD8^+^ T cells. **(a)** MAIT cells were assayed for the intracellular expression of granzyme B in the presence or absence of indicated stimuli (cytokines IL‐12 and IL‐18 (50 ng mL ^−1^) ± anti‐CD3/CD28 stimulation) in the presence or absence of 2‐deoxyglucose (2 mmol L^−1^). Significance was determined using a paired, parametric two‐tailed *t*‐test. Significance levels: * indicates *P* < 0.05, **P < 0.01, ***P < 0.001. Error bars show mean ± s.d. Cumulative data are shown for *n* = 3 healthy donors (representative of two independent experiments). **(b)** “Non‐MAIT” CD8^+^ T cells were assayed for the intracellular expression of granzyme B in the presence or absence of indicated stimuli (cytokines IL‐12 and IL‐18 (50 ng mL ^−1^) ± anti‐CD3/CD28 stimulation) in the presence or absence of 2‐deoxyglucose (2 mM). Significance was determined using a paired, parametric two‐tailed *t*‐test. Significance levels: * indicates *P* < 0.05, **P < 0.01, ***P < 0.001. Error bars show mean ± s.d. Data are shown for *n* = 3 healthy donors (representative of two independent experiments). **(c)** MAIT cells were assayed for the expression of CD69 in the presence or absence of indicated stimuli (cytokines IL‐12 and IL‐18 (50 ng mL ^−1^) ± anti‐CD3/CD28 stimulation) in the presence or absence of 2‐deoxyglucose (2 mmol L^−1^). Significance was determined using paired, parametric two‐tailed *t*‐test. Significance levels: * indicates *P* < 0.05, **P < 0.01, ***P < 0.001. Error bars show mean ± s.d. Data are shown for *n* = 3 healthy donors (representative of two independent experiments). **(d)** “Non‐MAIT” CD8^+^ T cells were assayed for the expression of CD69 in the presence or absence of indicated stimuli (cytokines IL‐12 and IL‐18 (50 ng mL ^−1^) ± anti‐CD3/CD28 stimulation) in the presence or absence of 2‐deoxyglucose (2 mmol L^−1^). Significance was determined using a paired, parametric two‐tailed *t*‐test. Significance levels: * indicates *P* < 0.05, **P < 0.01, ***P < 0.001. Error bars show mean ± s.d. Data are shown for *n* = 3 healthy donors (representative of two independent experiments).

Stimulation of CD8^+^ T cells via the TCR leads to the initiation of effector functions and is dependent on the upregulation of glucose uptake.^24^ We were thus interested in whether the increase in glucose uptake seen in MAIT cells correlated with enhanced upregulation of effector molecules. Indeed, after the stimulation of MAIT cells with cytokines, we observed an upregulation of granzyme B expression which was enhanced when both stimuli were combined (Figure 3a). In contrast, granzyme B expression was not affected at all following stimulation in the other CD8^+^ T cell subsets (Figure 3b). To further understand the role of glycolysis in an activated MAIT cell, the glucose analogue 2‐deoxyglucose, which is able to inhibit the glycolytic pathways in T cells through the inhibition of the rate limiting enzyme hexokinase, was used.^25^ We cultured cells in the presence of this agent and assessed the expression of granzyme B and CD69. A significantly lower expression of granzyme B was observed upon inhibition of the glycolytic pathway (Figure 3a) and we could show that this effect was dose‐dependent (Supplementary figure3a). In contrast, this was not the case in CD161^−^ CD8^+^ T cells and T cell subsets (Figure 3b and Supplementary figure 3b). When we replaced glucose in the medium with equimolar concentrations of galactose, we could not see a difference in upregulation of granzyme B despite galactose metabolism being transcriptionally enriched in CD161^++^CD8^+^ *versus* CD161^−^CD8^+^ T cells (Supplementary figures 1a, 3c).

Furthermore, we observed an upregulation of the cellular activation marker CD69 in both MAIT cells and “Non‐MAIT” CD8^+^ T cells which was predominantly dependent on stimulation of the T cell receptor (Figure 3c, d). The expression of CD69 upon stimulation was not impaired by inhibiting glycolysis (Figure 3c, d).

Taken together, these findings indicate that the upregulation of granzyme B in MAIT cells upon stimulation is dependent on the upregulation of the glycolytic pathway. This is similar to the increased effector functions seen in effector T cells with a corresponding initiation of glycolysis and inhibition of effector functions upon limiting glucose availability.

## Discussion

This study aimed to investigate the preferences of MAIT cells in their usage of metabolic pathways in a resting state and upon activation. We found that in the resting state, MAIT cells are metabolically quiescent, contain low levels of mitochondria, which are largely well‐polarized. Examining their respiratory capacity using metabolic flux analysis, MAIT cells showed a lower potential for maximal respiration compared to “Non‐MAIT” cells. It has been previously shown that *T*_N_ had a lower SRC compared to *T*_CM_ and *T*_EM_ 26 and that this could be due to a reduced number of mitochondria.^11^ Supporting this, Gene Set Enrichment Analysis revealed reduced expression of the oxidative phosphorylation gene set. Interestingly, the tendency toward more polarized and functional mitochondria, similar to what can be examined in *T*_N_ and *T*_CM_, is in contrast to the distinct “effector‐memory” profile of the MAIT cells in normal human blood.

*Ex vivo*, MAIT cells at a quiescent state consistently show a moderate amount of uptake of the fluorescent glucose analogue 2‐NBDG. Metabolic flux analysis revealed that their basal and maximal extracellular acidification rates are lower compared to “Non‐MAIT” CD8^+^ T cells. It has been previously shown that *T*_N_ and *T*_CM_ cells had a lower capacity for extracellular acidification compared to *T*_EM_ and *T*_EMRA._ 26 Therefore, resting MAIT cells resemble the naïve and central memory T cell subset in this respect, as well in functional responses—apoptosis and autophagy.

Glycolysis has been shown to be the predominant pathway used during T cell activation and effector function.^24^ Similarly, once MAIT cells are activated they increase their uptake of the fluorescent glucose analogue 2‐NBDG in a synergistic, stimulus dependent manner. The lowest uptake was observed with the combination of the cytokines IL‐12 and IL‐18, which increased with anti‐CD3/CD28 stimulation and was synergistically increased upon combining these two stimuli. It has been reported before that MAIT cells show low granzyme B expression at a basal level but that its expression can be induced upon T cell receptor stimulation and co‐culture with *E*. *coli* in a stepwise manner.^27^ Granzyme B is a key protein for efficient cytotoxic activity and it accumulates in cytotoxic T cells following stimulation. Interestingly, it is not constitutively expressed upon stimulation in MAIT cells unlike other CD8^+^ T cell subsets.^27^

Here, we provide further evidence that this process is likely dependent on a functioning glycolytic pathway as the upregulation of granzyme B was lost upon inhibition of glycolysis by 2‐deoxyglucose. In this respect, MAIT cells behave in a similar manner to naïve T cells which increase the use of their glycolytic pathway at their effector stage following antigen encounter. However, other effector functions of MAIT cells including the production of IFN‐γ, IL‐17, perforin and TNF‐α could be regulated independently from a functioning glycolytic pathway.

In summary, we have demonstrated a metabolic duality in MAIT cells. On the one hand they show characteristics of *T*_CM_ and naïve T cells at rest (in contrast to their *T*_EM_ profile) but on the other hand they resemble effector T cells following stimulation. This mode of action may reflect their role at the bridge between innate and adaptive immunity. For comparison, human and murine NK cells show glycolysis‐dependent functions including granzyme B upregulation^28^ as do subsets of innate lymphoid cells (ILC2).^29^ MAIT cells play an emerging role in different pathologies, with features similar to ILCs, NK cells and T cells in different settings. Furthermore definition of their metabolic traits *in vivo*, especially under conditions of inflammation and hypoxia could be of future interest including for therapeutic modulation of their functions.

## Methods

### Donors

Healthy donors were leukocyte cones (NHS Blood Services, Oxford, UK). All subjects were recruited in agreement with the local ethics committees of all participating institutions.

### Microarray and gene set enrichment analysis

Cell sorting, RNA extraction and microarray analysis of CD161^+^ subsets within CD8^+^ T cells was performed as previously described.^8^

Microarray data have been deposited on GEO under the series accession number GSE62099. A previously published dataset has been used for further comparisons (GSE23663).^14^ Gene set enrichment analysis was performed using Broad Institute Software v3.0 (http://www.broadinstitute.org/gsea).

### Flow cytometry and ICS

Staining with extracellular and intracellular markers are described in the Supplementary Information.

### *In vitro* metabolic assays

Mitochondrial mass and mitochondrial polarization was assessed by incubating 1 × 10^6^ PBMCs for 30 min at 37°C, 5% CO_2_ in full medium (RPMI 1640 (Life Technologies Ltd, Paisley, UK) supplemented with 10% FCS, Penicillin/Streptomycin, L‐Glutamine) and 12.5 nmol L^−1^ MitoTracker Green FM and MitoTracker Deep Red (Life Technologies Ltd) and by flow cytometry. To assess the uptake of the fluorescent glucose analog 2‐NBDG, cells were incubated for 30 min with 50 μmol L^−1^ 2‐NBDG (Cambridge Bioscience, Cambridge, UK) supplemented in glucose‐free RPMI 1640. Uptake of 2‐NBDG was measured by flow cytometry after surface staining. For more information, see Supplementary Information.

### Cell sorting and seahorse extracellular flux analysis

Human PBMCs were isolated from heparinized blood using Lymhoprep^TM^ (STEMCELL Technologies Ltd., Vancouver, Canada). 9.25 × 10^7^ and 2.5 × 10^8^ PBMCs of two donors were cultured in full medium for 20 h at 37°C 5%CO_2_, isolated using a magnetic CD8^+^ T Cell Isolation Kit (Miltenyi Biotec Ltd., Bergisch Gladbach, Germany), according to the manufacturer's instructions. For cell sorting, dead cells were excluded with the LIVE/DEAD™ Fixable Near‐IR Dead Cell Stain Kit (Life Technologies Ltd). CD8^+^ T cells were labeled with saturating concentrations of anti‐CD161 PE (Miltenyi Biotec Ltd.) and anti‐Vα7.2 PE (Biolegend, California, USA). Cells were sorted for both live CD161^++^V⍺7.2^+^ CD8^+^ T cells and CD161^−^ Vα7.2^−^ CD8^+^ T cells using a MoFlo™ (Beckman Coulter, Brea, California) cell sorter. The purity was over 95%.

Seahorse experiments were performed on sorted cells of two donors using the Seahorse XFp Cell Mito Stress Test Kit and the XFp Glycolysis Stress Test Kit (Agilent Technologies, California, USA). For more information, see Supplementary Information.

### *In vitro* stimulations

1 × 10^6^ PBMCs were stimulated for 20 h with recombinant human IL‐12 (50 ng mL^−1^) and IL‐18 (50 ng mL^−1^; MBL International, Woburn, USA) and/or anti‐CD3/CD28 beads (Miltenyi Biotec Ltd). To inhibit glycolytic metabolism 2‐deoxyglucose (Sigma‐Aldrich Company Ltd, Gillingham, UK) was added at a concentration of 2 mmol L^−1^ in glucose‐free RPMI (Life Technologies Ltd).

### Confocal microscopy

Sorted CD161^++^Vα7.2^+^ CD8^+^ T cells and CD161^−^ Vα7.2^−^ CD8^+^ T cells were incubated with 800 nmol L^−1^ MitoTracker Green FM and MitoTracker Deep Red (Life Technologies Ltd) supplemented medium for 30 min at 37°CO_2_ at 5%CO2 on coverslips. For more information, see Supplementary Information.

### Statistical analysis

All graphs and statistical analysis were performed using Prism v7 (GraphPad Software Inc., San Diego, USA). Statistical significance was assessed using a one‐way repeated measures ANOVA with Tukey's multiple comparison test or a paired *t*‐test.

## Author Contributions

MEZ and AJH performed the experiments. MEZ, AJH, BK, AK, CW, SJD and PK revised and finalized the manuscript. JH, TL, CP and CW helped with the data acquisition. EM performed the bioinformatics analysis. MEZ and AJH share co‐authorship for equal contributions to the design, execution and interpretation of the experiments.

## Conflict of Interest

The authors declare no conflicts of interest.
