# Supplementary material for: Human MAIT cells show metabolic quiescence with rapid glucose‐dependent upregulation of granzyme B upon stimulation
Source: Immunol Cell Biol. 2018 Mar 9;96(6):666–74. doi: 10.1111/imcb.12020 (PMC6055666; doi:10.1111/imcb.12020)
Supplement: Supplementary file 1 [file IMCB-96-666-s001.pdf]

Supplementary Figure S1A

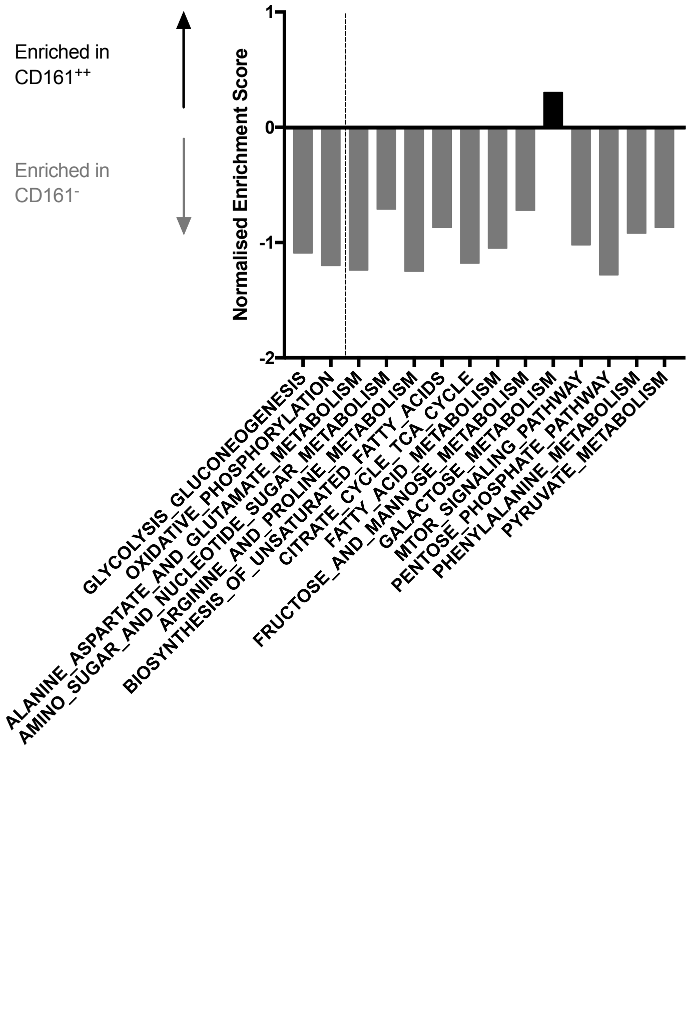

Supplementary Figure S1B

KEGG: OXPHOS

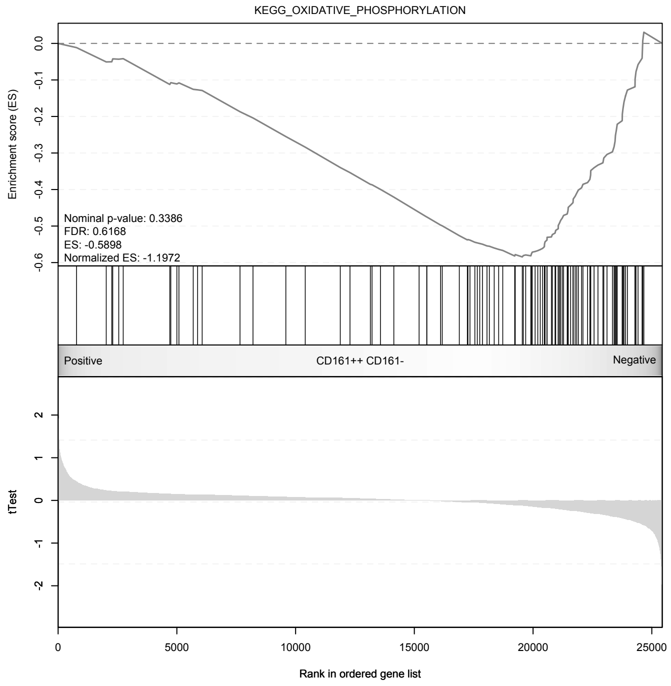

Supplementary Figure S1C

KEGG: Glycolysis

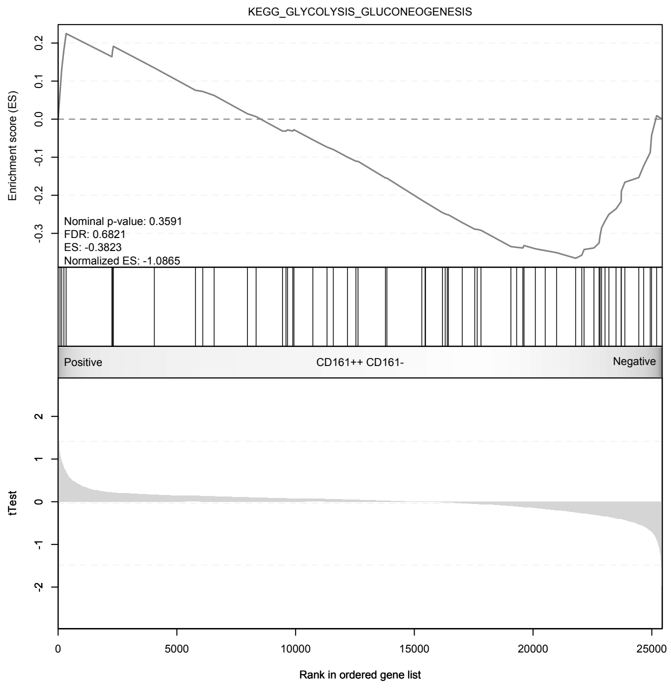

Supplementary Figure S1D

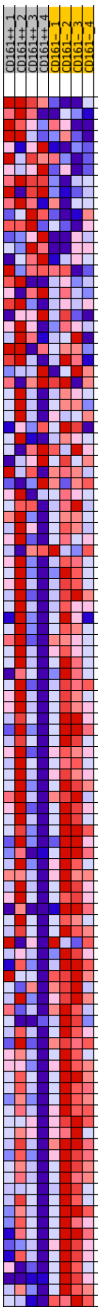

SampleName		
ATP6V0A1	ATP6V0A1	ATPase, H+ transporting, lysosomal V0 subunit a1
COX17	COX17	COX17 cytochrome c oxidase assembly homolog (S. cerevisiae)
ATP6V0B	ATP6V0B	ATPase, H+ transporting, lysosomal 21kDa, V0 subunit b
ATP6V0D1	ATP6V0D1	ATPase, H+ transporting, lysosomal 38kDa, V0 subunit d1
COX8C	COX8C	cytochrome c oxidase subunit 8C
COX7A1	COX7A1	cytochrome c oxidase subunit VIIa polypeptide 1 (muscle)
ATP6V1G3	ATP6V1G3	ATPase, H+ transporting, lysosomal 13kDa, V1 subunit G3
COX6B2	COX6B2	cytochrome c oxidase subunit VIb polypeptide 2 (testis)
ATP6V0A4	ATP6V0A4	ATPase, H+ transporting, lysosomal V0 subunit a4
ATP6AP1	ATP6AP1	ATPase, H+ transporting, lysosomal accessory protein 1
NDUFC2	NDUFC2	NADH dehydrogenase (ubiquinone) 1, subcomplex unknown, 2, 14.5kDa
ATP4A	ATP4A	ATPase, H+/K+ exchanging, alpha polypeptide
COX4I2	COX4I2	cytochrome c oxidase subunit IV isoform 2 (lung)
COX6A2	COX6A2	cytochrome c oxidase subunit VIa polypeptide 2
COX7B2	COX7B2	cytochrome c oxidase subunit VIIb2
NDUFS6	NDUFS6	NADH dehydrogenase (ubiquinone) Fe-S protein 6, 13kDa (NADH-coenzyme Q reductase)
ATP6V0C	ATP6V0C	ATPase, H+ transporting, lysosomal 16kDa, V0 subunit c
ATP6V1C2	ATP6V1C2	ATPase, H+ transporting, lysosomal 42kDa, V1 subunit C2
NDUFB7	NDUFB7	NADH dehydrogenase (ubiquinone) 1 beta subcomplex, 7, 18kDa
ATP12A	ATP12A	ATPase, H+/K+ transporting, nongastric, alpha polypeptide
ATP6V1A	ATP6V1A	ATPase, H+ transporting, lysosomal 70kDa, V1 subunit A
NDUFB1	NDUFB1	NADH dehydrogenase (ubiquinone) 1 beta subcomplex, 1, 7kDa
ATP6V1E1	ATP6V1E1	ATPase, H+ transporting, lysosomal 31kDa, V1 subunit E1
ATP6V1B2	ATP6V1B2	ATPase, H+ transporting, lysosomal 56/58kDa, V1 subunit B2
ATP6V1G2	ATP6V1G2	ATPase, H+ transporting, lysosomal 13kDa, V1 subunit G2
ATP6V0D2	ATP6V0D2	ATPase, H+ transporting, lysosomal 38kDa, V0 subunit d2
NDUFV2	NDUFV2	NADH dehydrogenase (ubiquinone) flavoprotein 2, 24kDa
ATP6V1D	ATP6V1D	ATPase, H+ transporting, lysosomal 34kDa, V1 subunit D
ATP6V0A2	ATP6V0A2	ATPase, H+ transporting, lysosomal V0 subunit a2
ATP5G3	ATP5G3	ATP synthase, H+ transporting, mitochondrial F0 complex, subunit C3 (subunit 9)
NDUFS7	NDUFS7	NADH dehydrogenase (ubiquinone) Fe-S protein 7, 20kDa (NADH-coenzyme Q reductase)
ATP4B	ATP4B	ATPase, H+/K+ exchanging, beta polypeptide
NDUFB4	NDUFB4	NADH dehydrogenase (ubiquinone) 1 beta subcomplex, 4, 15kDa
UQCRC1	UQCRC1	ubiquinol-cytochrome c reductase core protein I
ATP6V1C1	ATP6V1C1	ATPase, H+ transporting, lysosomal 42kDa, V1 subunit C1
NDUFV1	NDUFV1	NADH dehydrogenase (ubiquinone) flavoprotein 1, 51kDa
COX8A	COX8A	cytochrome c oxidase subunit 8A (ubiquitous)
ATP5J2	ATP5J2	ATP synthase, H+ transporting, mitochondrial F0 complex, subunit F2
NDUFB2	NDUFB2	NADH dehydrogenase (ubiquinone) 1 beta subcomplex, 2, 8kDa
CYC1	CYC1	cytochrome c-1
ATP6V1B1	ATP6V1B1	ATPase, H+ transporting, lysosomal 56/58kDa, V1 subunit B1 (Renal tubular acidosis with deafness)
NDUFA3	NDUFA3	NADH dehydrogenase (ubiquinone) 1 alpha subcomplex, 3, 9kDa
NDUFA11	NDUFA11	NADH dehydrogenase (ubiquinone) 1 alpha subcomplex, 11, 14.7kDa
SDHB	SDHB	succinate dehydrogenase complex, subunit B, iron sulfur (Ip)
ATP5E	ATP5E	ATP synthase, H+ transporting, mitochondrial F1 complex, epsilon subunit
ATP5I	ATP5I	ATP synthase, H+ transporting, mitochondrial F0 complex, subunit E
LOC644310	LOC644310	-
NDUFAB1	NDUFAB1	NADH dehydrogenase (ubiquinone) 1, alpha/beta subcomplex, 1, 8kDa
NDUFB10	NDUFB10	NADH dehydrogenase (ubiquinone) 1 beta subcomplex, 10, 22kDa
COX7B	COX7B	cytochrome c oxidase subunit VIIb
NDUFA4	NDUFA4	NADH dehydrogenase (ubiquinone) 1 alpha subcomplex, 4, 9kDa
PPA2	PPA2	pyrophosphatase (inorganic) 2
NDUFB8	NDUFB8	NADH dehydrogenase (ubiquinone) 1 beta subcomplex, 8, 19kDa
COX6A1	COX6A1	cytochrome c oxidase subunit VIa polypeptide 1
LHPP	LHPP	-
NDUFA1	NDUFA1	NADH dehydrogenase (ubiquinone) 1 alpha subcomplex, 1, 7.5kDa
COX6C	COX6C	cytochrome c oxidase subunit VIc
ATP6V0E1	ATP6V0E1	ATPase, H+ transporting, lysosomal 9kDa, V0 subunit e1
NDUFS8	NDUFS8	NADH dehydrogenase (ubiquinone) Fe-S protein 8, 23kDa (NADH-coenzyme Q reductase)
NDUFB6	NDUFB6	NADH dehydrogenase (ubiquinone) 1 beta subcomplex, 6, 17kDa
COX5A	COX5A	cytochrome c oxidase subunit Va
ATP5G1	ATP5G1	ATP synthase, H+ transporting, mitochondrial F0 complex, subunit C1 (subunit 9)
NDUFA7	NDUFA7	NADH dehydrogenase (ubiquinone) 1 alpha subcomplex, 7, 14.5kDa
NDUFA9	NDUFA9	NADH dehydrogenase (ubiquinone) 1 alpha subcomplex, 9, 39kDa
ATP5F1	ATP5F1	ATP synthase, H+ transporting, mitochondrial F0 complex, subunit B1
ATP5B	ATP5B	ATP synthase, H+ transporting, mitochondrial F1 complex, beta polypeptide
ATP5A1	ATP5A1	ATP synthase, H+ transporting, mitochondrial F1 complex, alpha subunit 1, cardiac muscle
COX4I1	COX4I1	cytochrome c oxidase subunit IV isoform 1
NDUFS3	NDUFS3	NADH dehydrogenase (ubiquinone) Fe-S protein 3, 30kDa (NADH-coenzyme Q reductase)
ATP5D	ATP5D	ATP synthase, H+ transporting, mitochondrial F1 complex, delta subunit
COX7A2	COX7A2	cytochrome c oxidase subunit VIIa polypeptide 2 (liver)
NDUFA8	NDUFA8	NADH dehydrogenase (ubiquinone) 1 alpha subcomplex, 8, 19kDa
ATP5L	ATP5L	ATP synthase, H+ transporting, mitochondrial F0 complex, subunit G
NDUFA5	NDUFA5	NADH dehydrogenase (ubiquinone) 1 alpha subcomplex, 5, 13kDa
ATP5J	ATP5J	ATP synthase, H+ transporting, mitochondrial F0 complex, subunit F6
TCIRG1	TCIRG1	T-cell, immune regulator 1, ATPase, H+ transporting, lysosomal V0 subunit A3
ATP5G2	ATP5G2	ATP synthase, H+ transporting, mitochondrial F0 complex, subunit C2 (subunit 9)
COX7C	COX7C	cytochrome c oxidase subunit VIIc
NDUFA10	NDUFA10	NADH dehydrogenase (ubiquinone) 1 alpha subcomplex, 10, 42kDa
NDUFV3	NDUFV3	NADH dehydrogenase (ubiquinone) flavoprotein 3, 10kDa
UQCRFS1	UQCRFS1	ubiquinol-cytochrome c reductase, Rieske iron-sulfur polypeptide 1
ATP6V1F	ATP6V1F	ATPase, H+ transporting, lysosomal 14kDa, V1 subunit F
COX15	COX15	COX15 homolog, cytochrome c oxidase assembly protein (yeast)
ATP5H	ATP5H	ATP synthase, H+ transporting, mitochondrial F0 complex, subunit d
COX10	COX10	COX10 homolog, cytochrome c oxidase assembly protein, heme A: farnesyltransferase (yeast)
SDHA	SDHA	succinate dehydrogenase complex, subunit A, flavoprotein (Fp)
NDUFS2	NDUFS2	NADH dehydrogenase (ubiquinone) Fe-S protein 2, 49kDa (NADH-coenzyme Q reductase)
NDUFB3	NDUFB3	NADH dehydrogenase (ubiquinone) 1 beta subcomplex, 3, 12kDa
UQCRH	UQCRH	ubiquinol-cytochrome c reductase hinge protein
COX6B1	COX6B1	cytochrome c oxidase subunit Vib polypeptide 1 (ubiquitous)
ATP6V1H	ATP6V1H	ATPase, H+ transporting, lysosomal 50/57kDa, V1 subunit H
COX5B	COX5B	cytochrome c oxidase subunit Vb
PPA1	PPA1	pyrophosphatase (inorganic) 1
NDUFS5	NDUFS5	NADH dehydrogenase (ubiquinone) Fe-S protein 5, 15kDa (NADH-coenzyme Q reductase)
ATP6V1G1	ATP6V1G1	ATPase, H+ transporting, lysosomal 13kDa, V1 subunit G1
NDUFB5	NDUFB5	NADH dehydrogenase (ubiquinone) 1 beta subcomplex, 5, 16kDa
ATP5C1	ATP5C1	ATP synthase, H+ transporting, mitochondrial F1 complex, gamma polypeptide 1
SDHC	SDHC	succinate dehydrogenase complex, subunit C, integral membrane protein, 15kDa
SDHD	SDHD	succinate dehydrogenase complex, subunit D, integral membrane protein
ATP5O	ATP5O	ATP synthase, H+ transporting, mitochondrial F1 complex, O subunit (oligomycin sensitivity conferring protein)
UQCRB	UQCRB	ubiquinol-cytochrome c reductase binding protein
NDUFS4	NDUFS4	NADH dehydrogenase (ubiquinone) Fe-S protein 4, 18kDa (NADH-coenzyme Q reductase)
ATP6V1E2	ATP6V1E2	ATPase, H+ transporting, lysosomal 31kDa, V1 subunit E2
NDUFC1	NDUFC1	NADH dehydrogenase (ubiquinone) 1, subcomplex unknown, 1, 6kDa
LOC642502	LOC642502	-
COX11	COX11	COX11 homolog, cytochrome c oxidase assembly protein (yeast)
UQCRC2	UQCRC2	ubiquinol-cytochrome c reductase core protein II
NDUFS1	NDUFS1	NADH dehydrogenase (ubiquinone) Fe-S protein 1, 75kDa (NADH-coenzyme Q reductase)

Supplementary Figure S1E

CD161^{hi} T_{EM} vs. CD161^{lo} T_{EM}

KEGG: OXPHOS

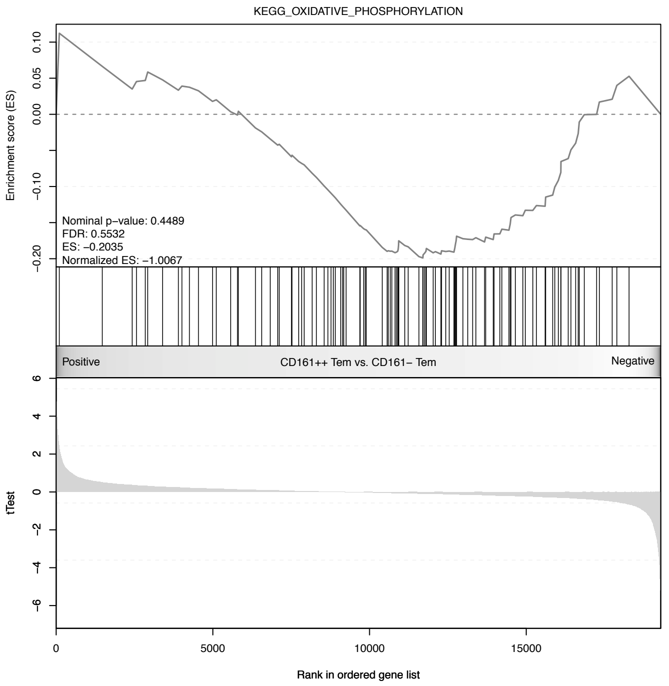

KEGG: Glycolysis

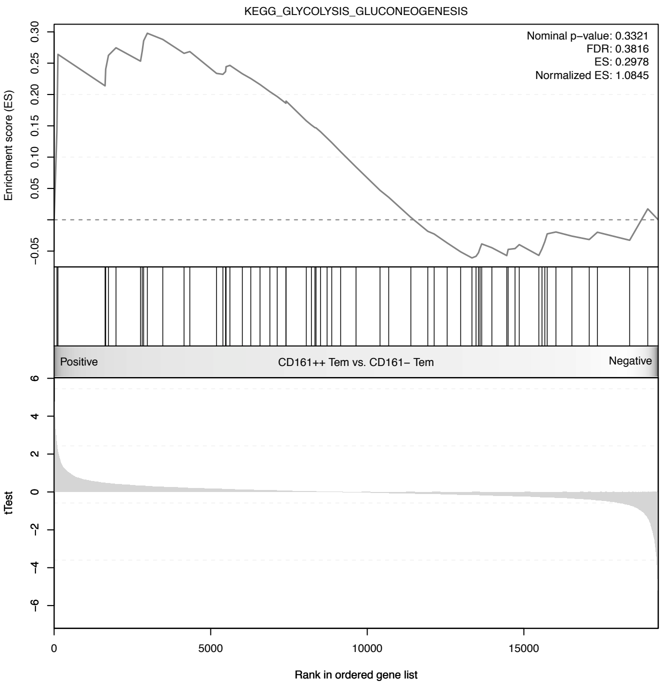

CD161^{hi} T_{EM} vs. CD161^{lo} T_{CM}

KEGG: OXPHOS

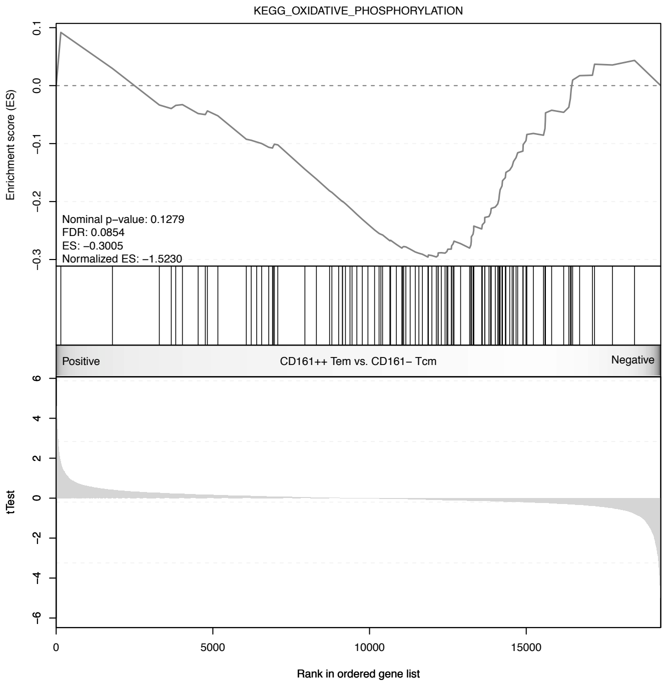

KEGG: Glycolysis

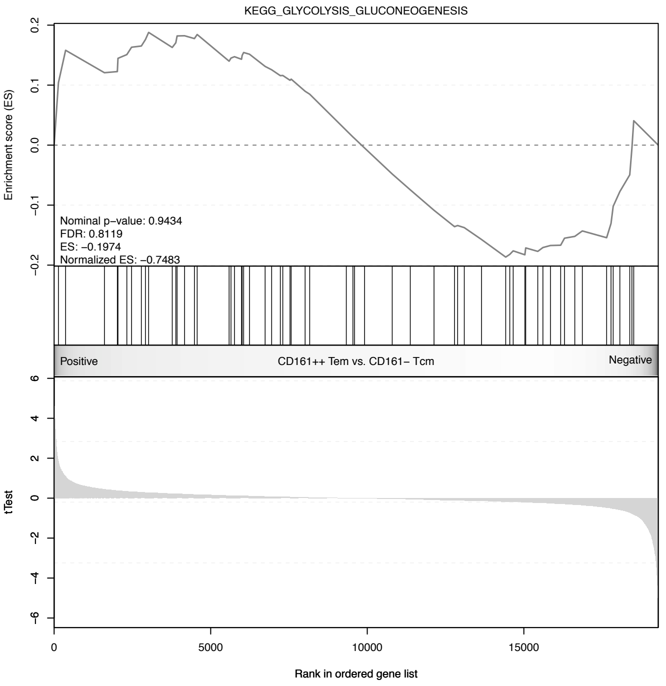

CD161^{hi} T_{EM} vs. T_N

KEGG: OXPHOS

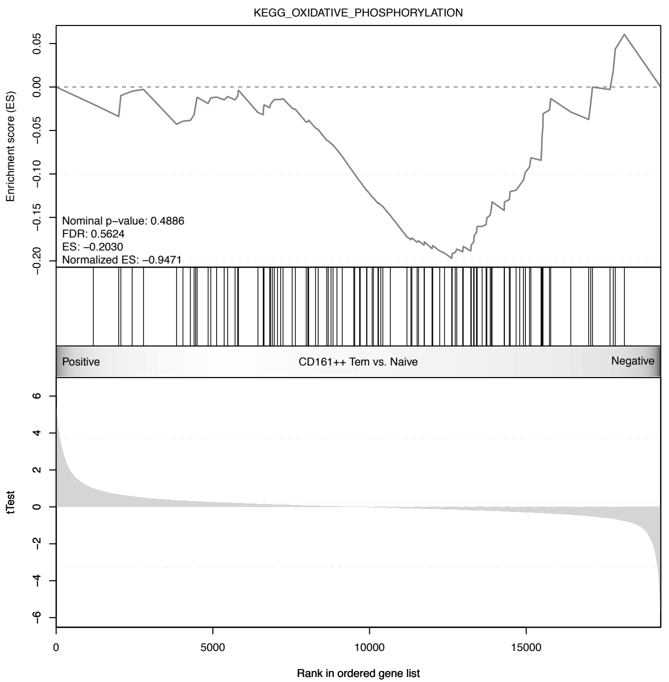

KEGG: Glycolysis

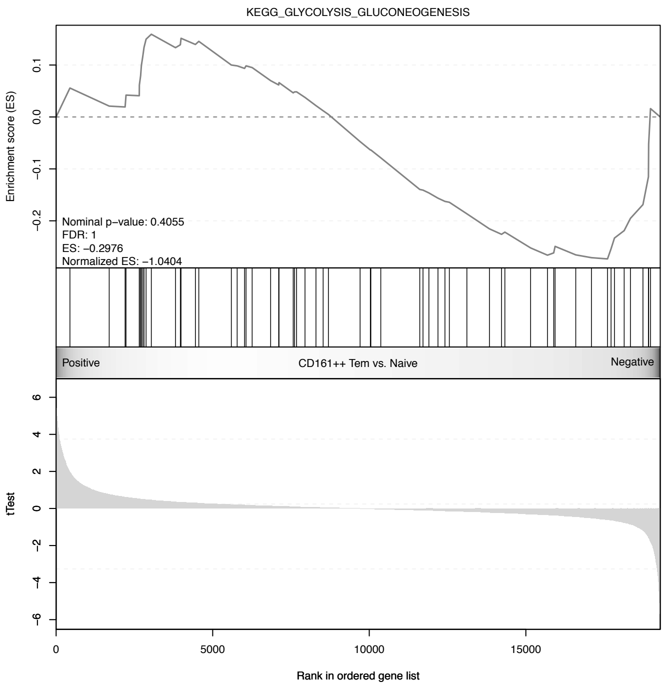

Supplementary Figure S2A

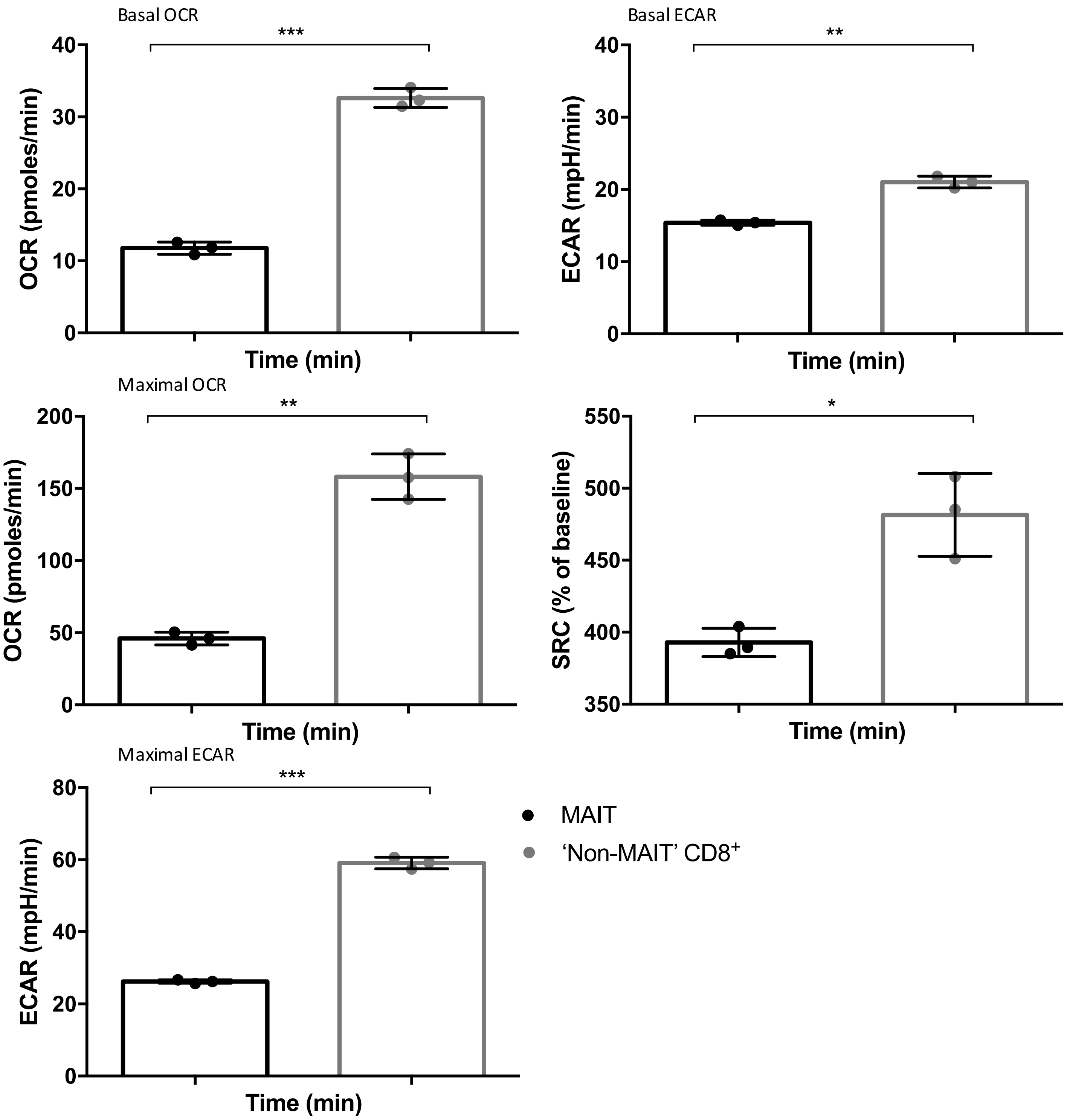

Supplementary Figure S2B

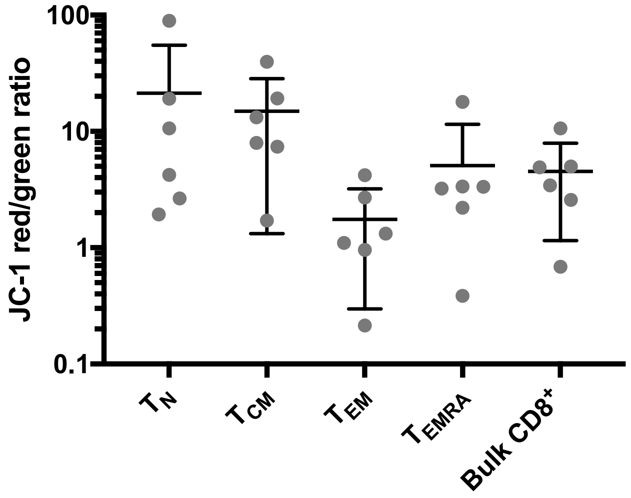

Supplementary Figure S2C

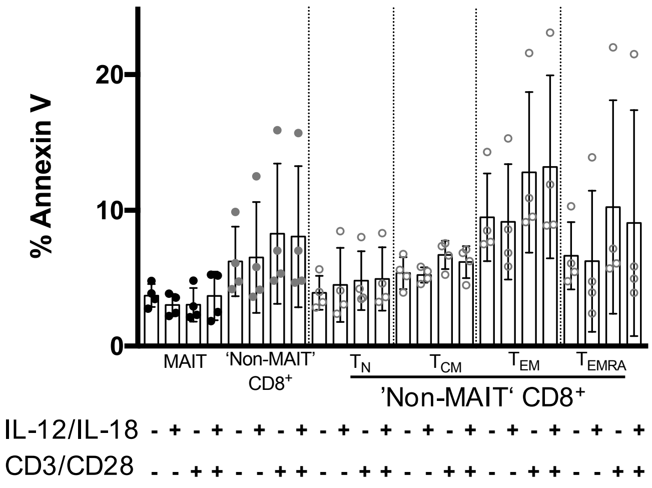

Supplementary Figure S2D

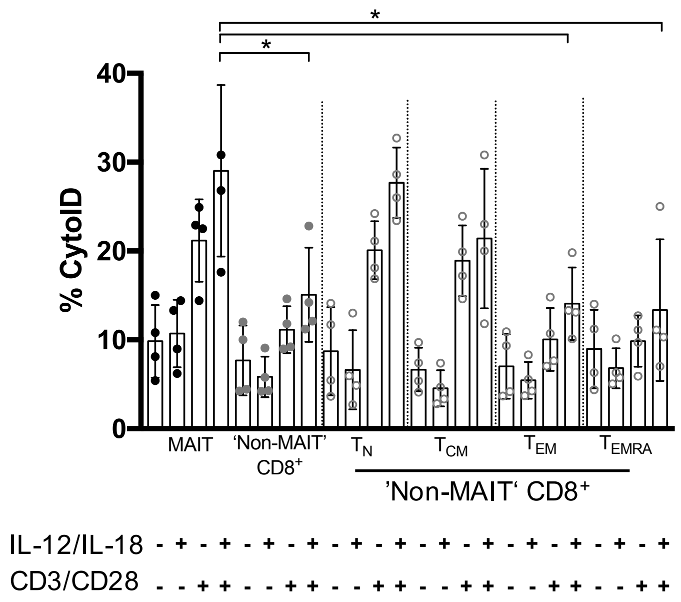

Supplementary Figure S3A

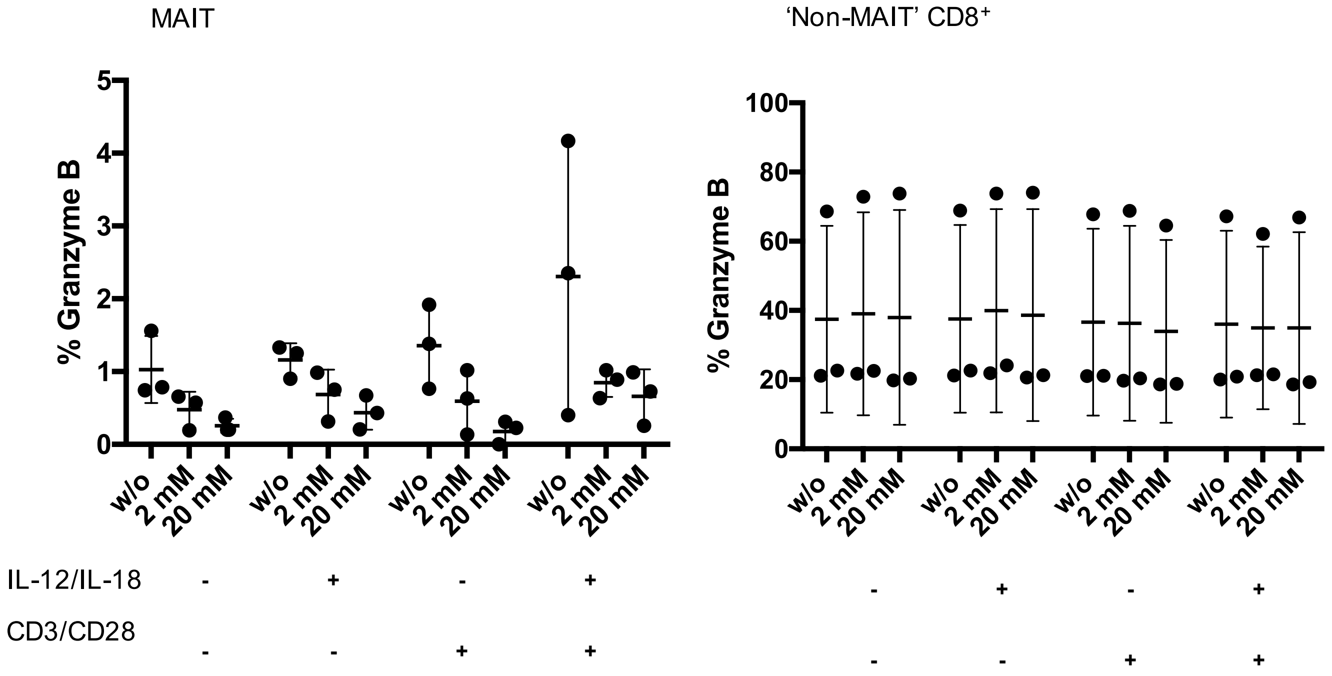

Supplementary Figure S3B

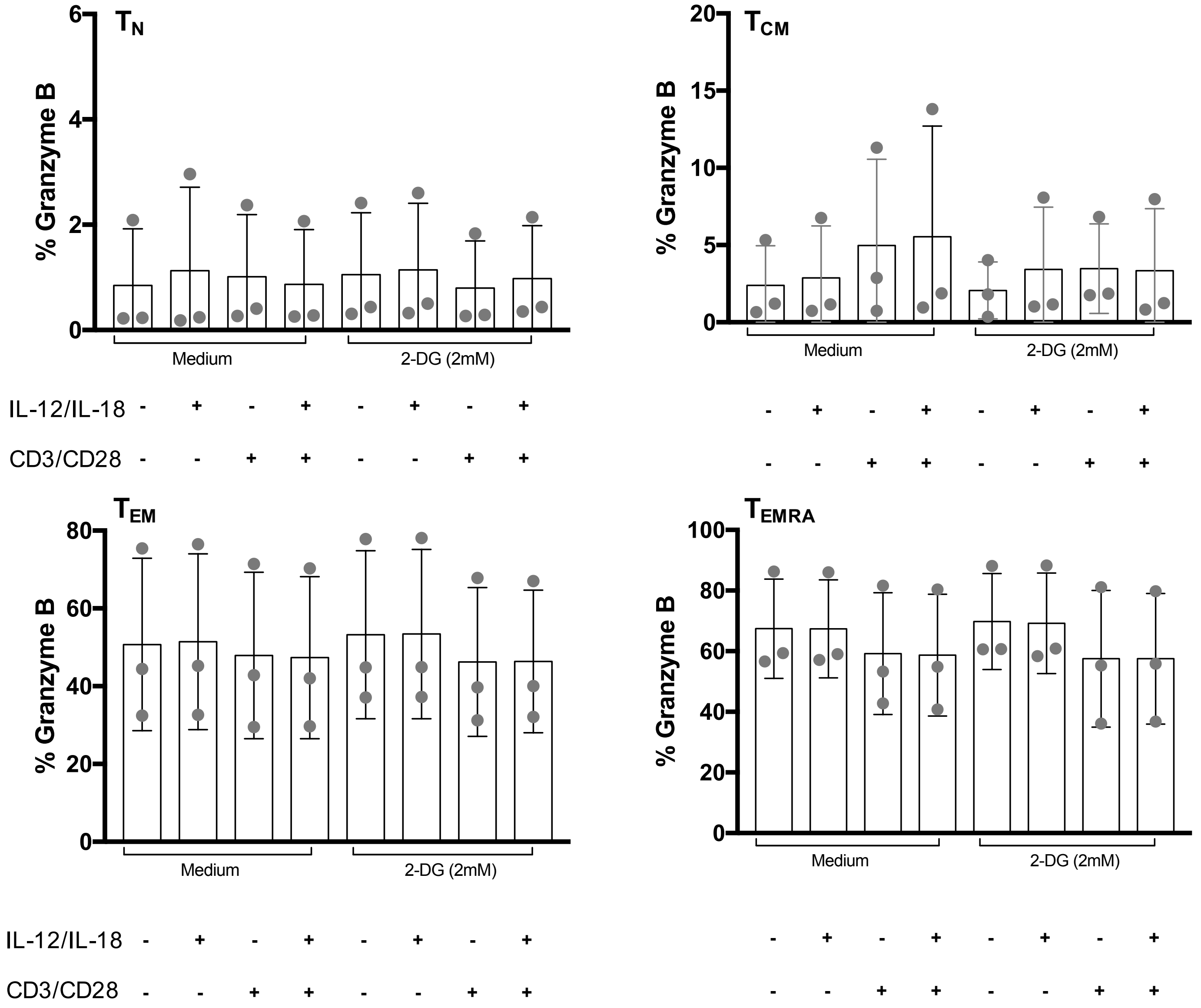

Supplementary Figure S3C

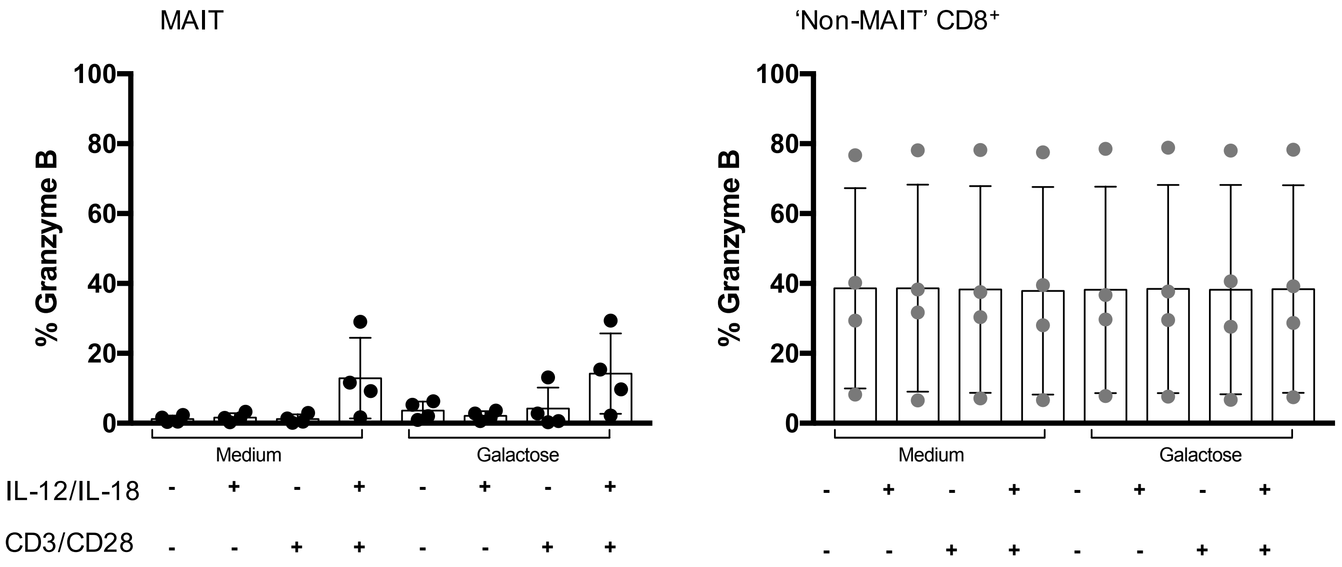

Supplementary Figure Legends

Supplementary Figure S1.

(A) Normalized enrichment scores (NES) for different metabolic gene sets comparing enrichment in CD161^{++} CD8^{+} T cells (black) to CD161^{-} CD8^{+} T cells (grey) based on microarray profiling of CD161^{++} CD8^{+} T cells or CD161^{-} CD8^{+} T cells. For the transcriptional analysis, sorted CD8^{+} T cells were used from four healthy donors.

(B) Enrichment plot of KEGG pathway for oxidative phosphorylation (OXPHOS) comparing CD161^{++} (left) to CD161^{-} (right). Negative normalized enrichment score (NES) indicates negative enrichment in CD161^{++} CD8^{+} T cells (NES=-1.1972).

(C) Enrichment plot of KEGG pathway for glycolysis and gluconeogenesis comparing CD161^{++} (left) to CD161^{-} (right). Negative normalized enrichment score (NES) indicates negative enrichment in CD161^{++} CD8^{+} T cells (NES=-1.0865).

(D) A heatmap of leading edge genes driving gene set enrichment of KEGG pathway of oxidative phosphorylation where the rank metric score of differential expression with the genes most upregulated in CD161^{++} CD8^{+} T cells vs. CD161^{-} CD8^{+} T cells ranked on top of the list.

(E) Enrichment plot of KEGG pathway for oxidative phosphorylation (OXPHOS; left) and glycolysis (right) comparing CD161^{hi} T_{EM} to CD161^{lo} T_{EM} (upper panels). Enrichment plot of KEGG pathway for oxidative phosphorylation (left) and glycolysis (right) comparing CD161^{hi} T_{EM} to CD161^{lo} T_{CM} (middle panels). Positive normalized enrichment score (NES) indicates enrichment in CD161^{hi} T_{EM} CD8^{+} T cells. Lower panels show enrichment plots for oxidative phosphorylation (left) and glycolysis (right) comparing CD161^{hi} T_{EM} and naïve CD8^{+} T cells. Data from 6

healthy individuals are shown (GEO: GSE23663).

Supplementary Figure S2.

(A) Seahorse metabolic profiling was assayed on isolated MAIT cells and 'Non-MAIT' $CD8^+$ T cells baseline oxygen consumption rate (OCR) and extracellular acidification rate (ECAR) were determined. Maximal OCR and spare respiratory capacity (SRC, expressed as the percentage of baseline) were measured upon injection of the mitochondrial uncoupling agent FCCP on the indicated cell types. Maximal ECAR was measured upon injection of oligomycin. Significance was determined using paired, parametric two-tailed t-test. Significance levels: * indicates $P < 0.05$, ** $P < 0.01$, *** $P < 0.001$. Error bars are a mean ± SD. Data from two healthy donors in triplicate wells is shown.

(B) Measurement of mitochondrial polarization status using the ratiometric dye JC-1. Results are expressed as ratio of red/green fluorescence with a higher ratio indicating more polarized mitochondria. Examined cell types were T_N, T_{CM}, T_{EM} and T_{EMRA} and bulk 'Non-MAIT' $CD8^+$ T cells. Data from six healthy donors is shown (representative of two independent experiments).

(C) Frequency of apoptotic cells measured by Annexin V staining of the indicated subsets: MAIT cells and 'Non-MAIT' $CD8^+$ T cells (comprising T_N, T_{CM}, T_{EM} and T_{EMRA}) upon stimulation. Data is shown from n=4 healthy donors.

(D) Levels of autophagy measured by CytoID staining comparing MAIT cells and 'Non-MAIT' $CD8^+$ T cells. Cells were either left untreated or stimulated with IL-12 and IL-18 (50 ng mL^{-1}) with or without stimulation with anti-CD3/CD28 beads as indicated. $CD161^-$ cells comprise T_N, T_{CM}, T_{EM} and T_{EMRA}.

Significance was determined using paired, parametric two-tailed t-test. Significance levels: * indicates $P < 0.05$, ** $P < 0.01$, *** $P < 0.001$. Error bars show mean ± SD. Data is shown from n=4 healthy donors (representative of two independent experiments).

Supplementary Figure S3.

(A) MAIT and 'Non-MAIT' CD8+ T cells were assayed for the intracellular expression of granzyme B in the presence or absence of the indicated stimuli (cytokines Il-12 and IL-18 (50 ng mL^{-1}) anti-CD3/CD28 stimulation) in the presence (2 mM or 20 mM) or absence of 2-deoxyglucose (w/o). Significance was determined using paired, parametric two-tailed t-test. Significance levels: * indicates $P < 0.05$, ** $P < 0.01$, *** $P < 0.001$. Error bars show mean ± SD. Data is shown for n=3 healthy donors.

(B) 'Non-MAIT' $CD8^+$ T cell subsets (T_N, T_{CM}, T_{EM} and T_{EMRA}) were assayed for the intracellular expression of granzyme B in the presence or absence of indicated stimuli (cytokines IL-12 and IL-18 (50 ng mL^{-1}) ± anti-CD3/CD28 stimulation) in the presence or absence of 2-deoxyglucose (2 mM). Significance was determined using paired, parametric two-tailed t-test. Significance levels: * indicates $P < 0.05$, ** $P < 0.01$, *** $P < 0.001$. Error bars show mean ± SD. Data is shown for n=3 healthy donors (representative of two independent experiments).

(C) MAIT and 'Non-MAIT' CD8+ T cells were assayed for the intracellular expression of granzyme B in the presence or absence of the indicated stimuli (cytokines IL-12 and IL-18 (50 ng mL^{-1}) ± anti-CD3/CD28 stimulation) either in regular medium or in galactose supplemented medium (10 mM).

Significance was determined using paired, parametric two-tailed t-test. Significance levels: * indicates *P* < 0.05, ** *P* < 0.01, *** *P* < 0.001. Error bars show mean ± SD. Data is shown from n=4 healthy donors (representative of two independent experiments).

Supplementary Methods

Gene Set Enrichment Analysis

For Gene Set Enrichment Analysis, we used gene sets from KEGG pathway gene lists (http://www.genome.jp/kegg/pathway.html). The obtained Normalized Enrichment Scores (NES) were used to compare KEGG pathways in $CD161^{++}CD8^{+}$ T cells with $CD161^{-}CD8^{+}$ T cells. Furthermore, comparisons between $C161^{++}$ $CD8^{+}$ T cells and $CD161^{-}$ T_{N}, T_{CM}, T_{EM} and T_{EMRA} $CD8^{+}$ T cells were carried out using published microarray datasets on $CD8^{+}$ memory subsets (T_{N} and T_{EMRA}) and $CD161^{-}$ T_{CM} and T_{EM}[1].

Flow cytometry and ICS

For immunofluorescence staining, dead cells were excluded with the LIVE/DEAD™ Fixable Near-IR Dead Cell Stain Kit (Life Technologies Ltd). Staining was performed with the following antibodies: anti-CD8 BV650, anti-Granzyme B AlexaFluor700 (BD Biosciences, Oxford, UK), anti-Vα7.2 PE or APC, anti-CD161 BV421, anti-CCR7 PerCP-Cy5.5, anti-CD69 FITC (BioLegend), anti-CD45RA PE-Cy7 (eBioscience, Life Technologies Ltd), anti-CD8 VioGreen (Miltenyi Biotec, Ltd.) and anti-CD27 FITC (BioLegend). Data were collected on a LSR II (BD Biosciences) and analyzed using FlowJo v.9.7.6 (BD Biosciences). In indicated experiments, staining for cellular markers was performed intracellularly using the Foxp3/Transcription Factor Staining Buffer Set (eBioscience, Life Technologies Ltd), according to the manufacturer's instructions.

Annexin V staining was used to assess frequencies of apoptotic cells using the APC Annexin V Apoptosis Detection Kit (BioLegend), according to the manufacturer's instructions after surface staining.

In vitro metabolic assays

Mitochondrial polarization was additionally determined by JC-1 (Life Technologies Ltd) incubation (2 µM) of labelled cells for 30 min at 37°C, 5% CO_2.

To assess mitochondrial ROS production, cells were first stained for surface markers and afterwards incubated with 5 µM MitoSOX Red (Life Technologies Ltd) for 30 min at 37°C, according to the manufacturer's instructions and then acquired on a LSR II flow cytometer (BD Biosciences).

Measurement of autophagic vacuoles was performed using the CYTO-ID® Autophagy detection kit (Enzo Life Sciences, Exeter, UK), according to the manufacturer's instructions. Surface staining was performed after incubation with the fluorescent dye and fluorescence acquired by flow cytometry.

Seahorse Extracellular Flux Analysis

Triplicates of 330,000 cells per well of $CD161^{++}V\alpha7.2^{+}$ CD8+ T cells and $CD161^{-}$ $V\alpha7.2^{-}$ $CD8^{+}$ T cells were plated on poly-D-lysine coated Seahorse XFp Cell Culture Miniplates, equilibrated for 30 min at 37°C. Before extracellular flux analysis, sorted cells were stimulated with 5 µg mL^{-1} plate bound anti-CD3 (OKT3, BioLegend) and 1 µg mL^{-1} soluble anti-CD28 (CD28.2, eBioscience, Life Technologies Ltd) for one hour at 37°C. For analysis of glycolytic capacity, cells were equilibrated in glucose-free medium according to the manufacturer's instructions. Cells were assayed for changes in oxidative consumption rate (OCR, pMoles/min) and extracellular acidification rate

(ECAR, mpH/min) after addition of oligomycin (1 µM), FCCP (0.5 µM) or antimycin A/Rotenone (0.5 µM) for Mito Stress Test and after addition of glucose (10 mM), oligomycin (1 µM) and 2-deoxyglucose (50 mM) for Glycolysis Stress Test.

Confocal Microscopy

For confocal microscopy, coverslips were coated with poly-D-lysine. After staining the cells on these coverslips, they were mounted onto microscope slides using VectaShield (Vector Laboratories Ltd., Peterborough, UK) and pictures acquired using a Zeiss LSM 880 Airyscan microscope, serial number 2802000323, (Helium-Neon Laser, 633 nm; GaAsP detector) using ZEN black software (Carl Zeiss Ltd, Oberkochen, Germany). Images were acquired at 16 bit. The pictures were processed using ImageJ v2.0.0 (www.imagej.net) without manipulation of threshold or signal ranges.

References

1 Turtle CJ, Delrow J, Joslyn RC *et al.* Innate signals overcome acquired TCR signaling pathway regulation and govern the fate of human CD161hi CD8α+ semi-invariant T cells. *Blood* 2011; **118**: 2752–2762.
