# Supplementary material for: Human MAIT cells show metabolic quiescence with rapid glucose‐dependent upregulation of granzyme B upon stimulation
Source: Immunol Cell Biol. 2018 Mar 9;96(6):666–74. doi: 10.1111/imcb.12020 (PMC6055666; doi:10.1111/imcb.12020)
Supplement: Supplementary file 2 [file IMCB-96-666-s002.docx]

**Summary of Contents of Supplementary Information**

1. Supplementary Figures: Figures providing additional information (.pdf)
2. Supplementary Figure Legends: Brief description of data and analyses performed in Supplementary Figures (.pdf)
3. Supplementary Methods: Additional information on methods applied (.pdf)
